# Single-cell RNA sequencing highlights the role of PVR/PVRL2 in the immunosuppressive tumour microenvironment in hepatocellular carcinoma

**DOI:** 10.3389/fimmu.2023.1164448

**Published:** 2023-06-13

**Authors:** Ang Li, Bai Ji, Yongsheng Yang, Bicheng Ye, Qinmei Zhu, Xintong Hu, Yong Liu, Peiwen Zhou, Juanjuan Liu, Ranran Gao, Qi Zhou, Boxi Kang, Yanfang Jiang

**Affiliations:** ^1^ Key Laboratory of Organ Regeneration and Transplantation of the Ministry of Education, Genetic Diagnosis Center, the First Hospital of Jilin University, Changchun, China; ^2^ Department of Hepatobiliary and Pancreatic Surgery, the First Hospital of Jilin University, Changchun, China; ^3^ Department of Hepatobiliary and Pancreas Surgery, the Second Hospital of Jilin University, Changchun, China; ^4^ School of Clinical Medicine, Medical College of Yangzhou Polytechnic College, Yangzhou, China; ^5^ Department of Bioinformatics, Analytical Biosciences Limited, Beijing, China

**Keywords:** single-cell RNA sequencing, HCC, TME, immunotherapy, PVR/PVRL2

## Abstract

**Introduction:**

The conflict between cancer cells and the host immune system shapes the immune tumour microenvironment (TME) in hepatocellular carcinoma (HCC). A deep understanding of the heterogeneity and intercellular communication network in the TME of HCC will provide promising strategies to orchestrate the immune system to target and eradicate cancers.

**Methods:**

Here, we performed single-cell RNA sequencing (scRNA-seq) and computational analysis of 35786 unselected single cells from 3 human HCC tumour and 3 matched adjacent samples to elucidate the heterogeneity and intercellular communication network of the TME. The specific lysis of HCC cell lines was examined in vitro using cytotoxicity assays. Granzyme B concentration in supernatants of cytotoxicity assays was measured by ELISA.

**Results:**

We found that VCAN+ tumour-associated macrophages (TAMs) might undergo M2-like polarization and differentiate in the tumour region. Regulatory dendritic cells (DCs) exhibited immune regulatory and tolerogenic phenotypes in the TME. Furthermore, we observed intensive potential intercellular crosstalk among C1QC+ TAMs, regulatory DCs, regulator T (Treg) cells, and exhausted CD8+ T cells that fostered an immunosuppressive niche in the HCC TME. Moreover, we identified that the TIGIT-PVR/PVRL2 axis provides a prominent coinhibitory signal in the immunosuppressive TME. In vitro, antibody blockade of PVR or PVRL2 on HCC cell lines or TIGIT blockade on immune cells increased immune cell-mediated lysis of tumour cell. This enhanced immune response is paralleled by the increased secretion of Granzyme B by immune cells.

**Discussion:**

Collectively, our study revealed the functional state, clinical significance, and intercellular communication of immunosuppressive cells in HCC at single-cell resolution. Moreover, PVR/PVRL2, interact with TIGIT act as prominent coinhibitory signals and might represent a promising, efficacious immunotherapy strategy in HCC.

## Introduction

Liver cancer is one of most prevalent malignancies in the world, with an estimated 905,677 new cases and 830,180 deaths occurring per year (1). Hepatocellular carcinoma (HCC) accounts for approximately 90% of liver cancers and has been closely related to hepatitis B virus (HBV) infection in China (1, 2). Due to poor liver function or distant metastasis, over 80% of HCC patients fail to meet surgical resection criteria (3). Three types of FDA-approved drugs, that is, tyrosine kinase inhibitors (TKIs) (sorafenib, regorafenib, cabozantinib, and lenvatinib), immune checkpoint inhibitors (ICIs) (nivolumab and pembrolizumab), and vascular endothelial growth factor inhibitors (bevacizumab) have been used for advanced HCC (4). However, the overall 5-year survival rate of HCC is still poor (5). As such, understanding relevant mechanisms and designing new treatment strategies for HCC have remained challenging tasks.

The tumor microenvironment (TME) has been a popular area for drug discovery (6). Unlike tumor cells, immune cells within the TME are genetically stable and thus are popular therapeutic targets with a reduced risk of resistance and tumor recurrence (7–9). These immune cells within TME are highly heterogeneous, with tumor-antagonizing or tumor-promoting functions (10). In the early stage of tumorigenesis, the tumor-antagonizing immune cells tend to target and kill the cancer cells. However, the cancer cells can eventually escape from immune surveillance and even suppress the cytotoxic function of tumor-antagonizing immune cells. Thus, a deep understanding of the immune cells in TME based on single-cell RNA sequencing (scRNA-seq) will provide promising strategies to target and eradicate cancers. Two recent studies demonstrated that T cells and immune cells exhibit various states in HCC using single-cell transcriptome analysis (11, 12). Given the limitations of functional analysis of immunosuppressive cells and intercellular crosstalk in the TME at the single-cell level, we performed scRNA-seq to better elucidate the mechanisms of HCC progression and with an aim to modulate the immune system to target and eradicate cancers.

In the current study, we profiled the transcriptome of 35786 single cells from HCC patients and matching nonmalignant tissue, and we provide a comprehensive global view of the multifaceted immunosuppressive landscape and their interactome in HCC TME that foster an immunosuppressive niche. More importantly, the TIGIT-PVR/PVRL2 axis was found to provide a prominent coinhibitory signal in the immunosuppressive TME. We show that blocking the TIGIT-PVR/PVRL2 axis enhanced immune cell-mediated lysis of HCC cells.

## Materials and methods

### Patients and samples

Three human HCC tumor and three matched adjacent samples that were surgically resected were selected randomly with the following criteria: (1) postoperative pathological diagnosis of HCC; (2) more than 200000 single cells in the dissociated suspension in each case; and (3) a dissociated single-cell viability rate of more than 85%. The human HCC tumor and adjacent samples were obtained immediately after surgical resection at the First Hospital of Jilin University. The study design was in accordance with the guidelines of the Declaration of Helsinki and was approved by the Human Ethics Committee of the First Hospital of Jilin University (21K089-001). Informed consent was obtained from all participants.

### Tissue dissociation and single-cell suspension preparation

Fresh tumor and adjacent normal tissue were processed immediately with mechanical dissociation and enzymatic digestion to generate single-cell suspensions. Briefly, each sample was cut into approximately 1-mm^3^ pieces in culture medium (DMEM; Gibco) with 10% fetal bovine serum (FBS; Gibco) and then incubated with type II (Thermo Fisher) and IV (Thermo Fisher) enzyme solution for 30 min on a 37°C shaker. The suspended cells were subsequently passed through 40 μm cell strainers (BD) and centrifuged at 400 g for 5 min. After the supernatant was discarded, the pelleted cells were suspended in 0.8% NH4Cl red blood cell lysis buffer and incubated on ice for 10 min to lyse red blood cells. After washing twice with DPBS (Gibco), the cell pellets were resuspended in sorting buffer containing 1X DPBS with 0.04% BSA (Sigma–Aldrich). At least 200,000 cells were collected from each sample.

### Droplet-based scRNA-seq

Chromium Single Cell 5′ Reagent kits were used according to the manufacturer’s instructions to construct barcoded scRNA-seq libraries. Briefly, the sorted cells were washed twice with sorting buffer. Then, a trypan blue (Thermo Fisher) staining exclusion assay was used to determine the cell viability and cell number. The single-cell suspension was further mixed with barcoded gel beads on a Chromium Controller (10x Genomics) to produce gel beads in emulsion (GEMs). To capture a target of 8000 cells per library, approximately 12,000 cells were loaded in each channel. After construction of GEMs, reverse transcription reactions were conducted to generate barcoded full-length cDNA, and amplification for 14 cycles was conducted on a thermal cycler (Bio–Rad). According to the instructions, cDNA sequencing libraries were constructed. The Qsep100 (Bioptic) analyzer was used to quantitate the average fragment size of a library. Every library was sequenced on a HiSeq X system (Illumina), and 150 bp paired-end reads were generated.

### Raw data processing and identification of cell types

Bcl2fastq (version v2.19.0.316, Illumina) was used to convert raw data from binary base call (BCL) format to FASTQ files. Sequencing reads in the FASTQ files were aligned to reference genomes of interest by using Cell Ranger pipelines (version 3.0.1; 10x Genomics) to subsequently generate feature-barcode matrices. Single-cell 5’-gene expression data were processed using Cell Ranger Count implemented in the pipelines. The gene expression data were mapped to the human genome reference sequence (GRCh38; http://cf.10Xgenomics.com/supp/cell-exp/refdata-cellranger-GRCh38-1.2.0.tar.gz). Then, the R package “DoubletFinder” (https://github.com/chris-mcginnisucsf/DoubletFinder) was applied to remove doublets in each sample individually. The filtered remaining cells were single cells. Next, the gene expression matrices of all remaining tumor and adjacent normal tissue cells were combined and converted into a Seurat object by using the R package Seurat (version 2.3.4, https://satijalab.org/seurat). Cells with less than 1001 unique molecular identifiers (UMIs), less than 501 genes, or over 10% mitochondrial-derived UMI counts were considered low-quality cells and were removed. Ultimately, 35786 single cells remained, and they were used for subsequent analyses. From the remaining cells, gene expression matrices were log normalized and subjected to linear regression by using the R Seurat package.

Since samples from the patients were processed independently and sequenced in batches, we used canonical correlation analysis (CCA) and the RunUMAP function implemented in Seurat to reduce the dimensionality of the scRNA-seq dataset and remove potential batch effects. The main cell clusters were identified with the FindClusters function in Seurat, with the resolution set as 0.5. Then, cell clusters were visualized with 2D UMAP plots. The average gene expression of conventional markers, described in a previous study was used to annotate the clusters as major biological cell types. First, 35786 cells were clustered into six major cell types. Subsequently, the abovementioned normalization, dimensionality reduction, and clustering processes were repeated, and the major cell types were further clustered into subclusters of different specific cell subtypes. The Seurat FindAllmarkers function was employed to identify preferentially expressed genes in each subcluster compared with the other subclusters.

### Pathway analysis and definition of signature scores

Pathway activities score of per cell implemented in the GSVA package (13) was used for enrichment analysis. Then, the differences in pathway activities scored per cell between C1QC+ TAMs and VCAN+ TAMs were calculated by two-sided unpaired limma-moderated t test. For three subclusters of DC cells (Regulatory DCs, cDC1s, and cDC2s), the differences in pathway activities scored per cell between regulatory DCs VS cDC1s & cDC2s; cDC1s VS regulatory DCs & cDC2s; cDC2s VS regulatory DCs & cDC1s were calculated by two-sided unpaired limma-moderated t test respectively. Then, the p value is adjusted by Benjamini-Hochberg method.

To calculate the M1/M2 score of TAMs and the antigen processing and presentation and immune regulatory scores of DCs, GSVA was conducted. Gene sets associated with the above functions were retrieved from previous studies (14, 15).

### Public dataset validation

The RNA sequencing transcriptome data of HCC patients were obtained from the TCGA database (https://portal.gdc.cancer.gov/), and the dataset contained 369 tumor samples and 50 non tumor samples. For further analysis, clinical data, including patient age, sex, grade, and TNM stage, were also downloaded.

For cell subgroup analysis, marker genes were defined with a cutoff of fold change (FC) >2, and the cell type signature values were defined as the log2 transformed mean TPM values of the marker genes. Correlations between specific cell types were estimated by Spearman correlation methods.

The relative abundances of cell types identified in this study were evaluated by CibersortX (16). Subsequently, all the TCGA LIHC patients were divided into high and low groups based on the optimal cutoff point, which was determined by the “survminer” R package. The prognostic significance of the relative abundance of cell types between the high and low groups was calculated by Kaplan–Meier curves. Multivariate Cox regression analysis confirmed that the relative abundance was an independent prognostic factor.

### Construction of single-cell trajectories

To characterize the potential translational relationships and lineage differentiation in the TME, we conducted trajectory analyses using Monocle2 (version 2.8.0; http://cole-trapnell-lab.github.io/monocle-release/monocle2/). A series of genes with key roles in differentiation were revealed by the Monocle2 plot_pseudotime_heatmap function. The top 1,000–2,000 significantly changed genes with a q-value < 0.01 were identified with the Monocle2 differential GeneTest function. After dimension reduction and cell ordering with the default parameters in Monocle2, the differentiation trajectory was inferred.

### Simultaneous gene regulatory network analysis

SCENIC is a new computational method used in the identification of different cell states and context-specific network models of transcriptional regulation networks from scRNA-seq data (17). The preferentially expressed regulons were calculated based on the differential expression of transcription factors or their target genes between cell clusters by using the wilcox test in seurat package. Regulons significantly upregulated or downregulated with adj. P < 0.05 in at least one cluster were assessed in further analysis.

### Cellular communication analysis

To investigate intercellular communication at the molecular level, we performed ligand–receptor analyses using the Python-based computational analysis tool CellPhoneDB. Ligand–receptor pairs with P < 0.05 returned by CellPhoneDB were selected for the evaluation of interactions between cell types.

### Cell lines and cell culture

The HCC cell lines, HepG2.2.15 and MHCC-LM3 were cultured in a humidified incubator with 37°C and 5% CO2 in DMEM (Gibco) supplemented with 1% penicillin-streptomycin and 10% FBS Superior (Biochrome GmbH), and were tested for mycoplasma contamination by using MycoAlert (Lonza).

### Cytotoxicity assays

To analyze the specific lysis of HCC cancer cells, target cells were stained with 5μM CellTracker™ Green CMFDA Dye (YEASEN). Healthy donor peripheral blood mononuclear cells (PBMCs) were isolated by density-gradient centrifugation using Lymphoprep™ (STEMCELL Technologies) from the Physical Examination Center of the Hospital. Labeled target cells and HD-PBMCs mixed in a ratio of 6:1 at 1 × 10^6^ cells/mL in the cell culture medium used to cultivate the target cell line. Two hundred microliter of cell suspension was plated in triplicates in 96-well plates incubated with or without Anti-PVR (10 µg/mL, Biolegend) (18), anti-PVRL2 (10 µg/mL, Biolegend) (18) or anti-TIGIT (50 µg/mL, Biolegend) (19). After 24 h of incubation, the remaining live target cells were assessed by measuring FVS780 (BD Biosciences) staining of gated CMFDA negative cells in flow cytometry. All experiments were conducted at least three times.

### Granzyme B ELISA

According to manufacturer’s instructions, the granzyme B concentration in supernatants of cytotoxicity assays was measured by using the human granzyme B DuoSet ELISA (R&D Systems).

### Generation of knockout cell lines using CRISPR/Cas9

Using CRISPR/Cas9 delivered by non-integrating lentiviral vectors (NILV), PVR and PVRL2 double knockout cell line were generated step wisely. Guide RNAs were designed using crispr.mit.edu (guide PVR GATGTTCGGGTTGCGCGTAG; guide PVRL2 CGGCGATCTCGACGGCAGGA). Clone the guide sequences into a lentiviral construct U6-cgRNA-SFFV-Cas9-IRES-mCherry, derived from pX330 and LeGO-iC (www.addgene.org) (20). As described previously 3rd-generation NILV were generated by using the packaging plasmids pRSV-Rev, phCMV-VSV-G and pCMVD8.74D64V (www.addgene.org) (21). Target cell lines were transduced with vector-containing supernatant and sorting of PVR/PVRL2 negative cells by flow cytometry.

### Statistical analysis

All statistical analyses and graph generation employed R software (version 3.6.0). Statistical significance was set at P<0.05.

## Results

### ScRNA-seq of HCC and matched nonmalignant tissues

**Figure 1:**
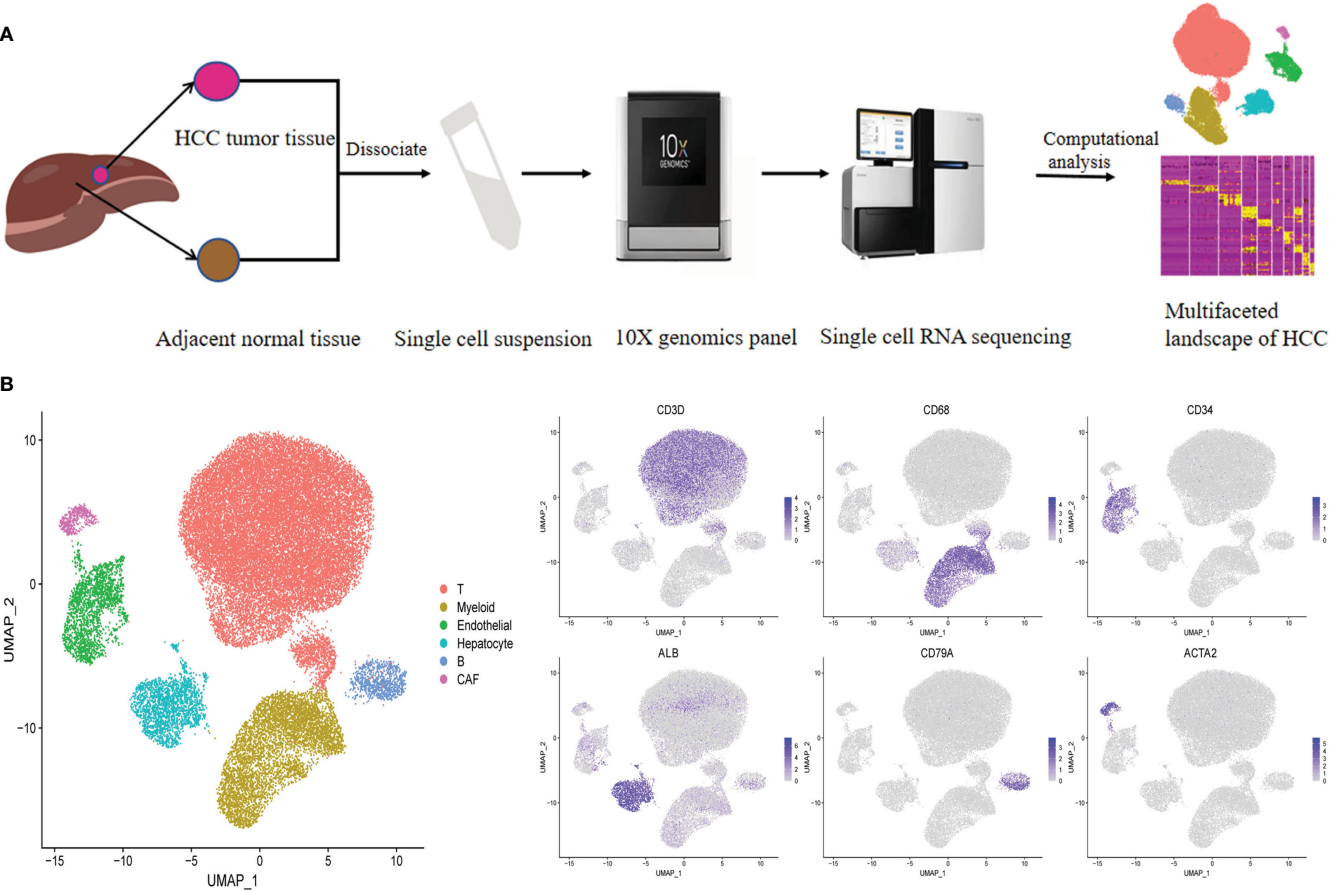
Identifying infiltrated cell type in HCC and matching non-malignant tissue. **(A)** Overall study design of experimental workflow, including sample preparation, high throughput sequencing and bioinformatics analysis. **(B)** UMAP plot of single cells colored by major cell type (left panel) and the expression of maker genes for major cell types (right panel). T represent tumor sample, P represent matching non-malignant sample.

To characterize the complexity of the TME in HCC, we employed scRNA-seq of infiltrated cell types derived from 3 HBV-positive human HCC tumor and 3 matched nonmalignant tissue samples taken prior to any anticancer treatment (**Figure 1A**; **Supplementary Table 1**). After strict quality control and removal of the batch effect between batches (**Supplementary Figures 1A–C**, see “Methods”), 35786 single cells were clustered into six major clusters via unsupervised clustering analysis implemented in Seurat software (**Figure 1B**); of the cells, 16211 and 19575 cells were derived from the tumor and matched nonmalignant tissue samples, respectively (**Supplementary Figure 1D**). The cells were further annotated as specific cell type subpopulations according to the expression of classic markers, including T cells (*CD3D+*), myeloid cells (*CD68+*), endothelial cells (*CD34+*), hepatocytes (*ALB+*), B cells (*CD79A+*), and cancer-associated fibroblasts (CAFs) (*ACTA2+*) (**Figure 1B**; **Supplementary Figure 1E, F**).

### VCAN+ TAMs undergo M2-like polarization in the tumor region

**Figure 2:**
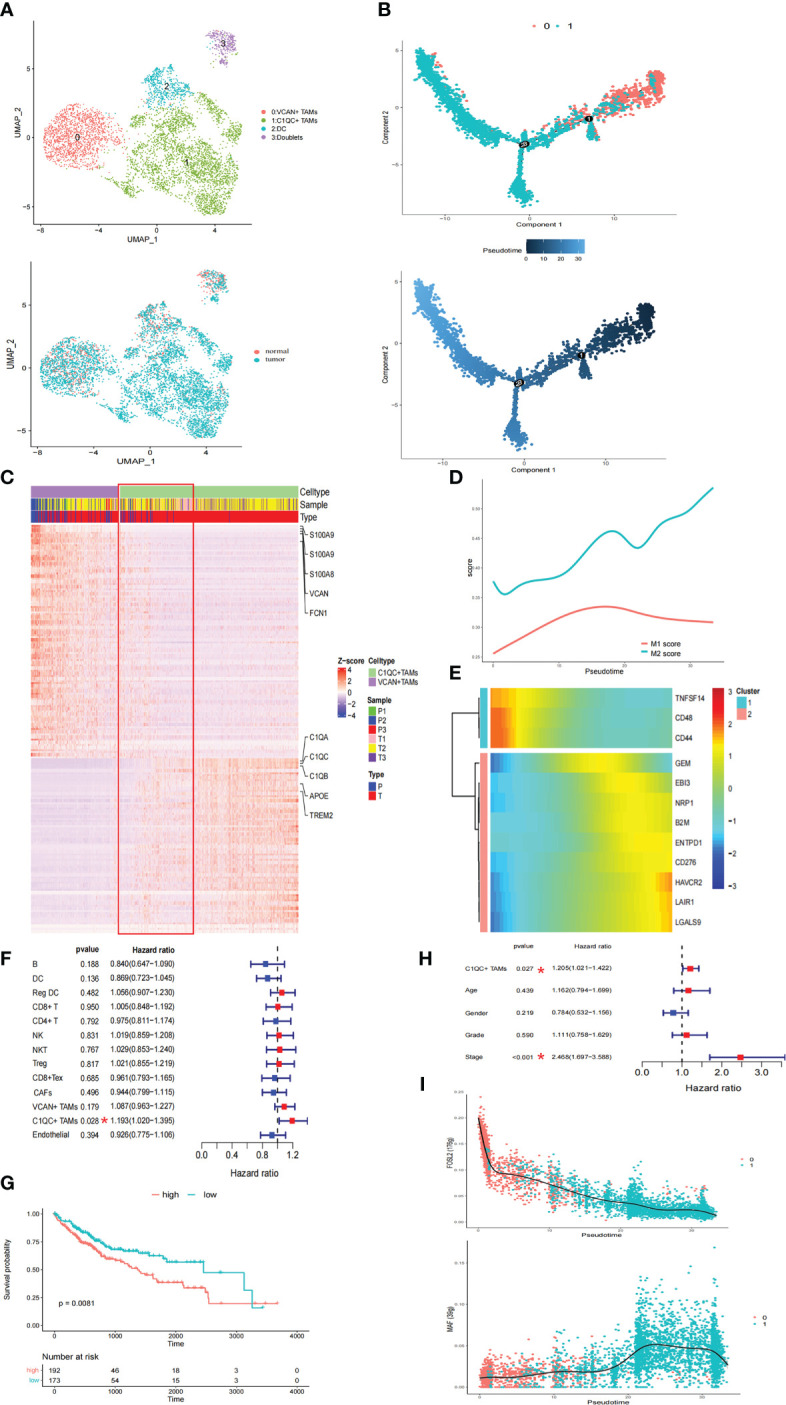
The TAMs in HCC exhibited a remarkable dichotomy. **(A)** UMAP plot of myeloid cells. **(B)** Trajectory of differentiation from VCAN+ TAMs to C1QC+ TAMs predicted by monocle. **(C)** DEGs between VCAN+ TAMs and C1QC+ TAMs. **(D)** M1 and M2 score in the differentiation process. **(E)** Heatmap show downregulated and upregulated immune checkpoints in the differentiation process. **(F)** Fraction of cell types and patient survival in samples from the TCGA LIHC cohort (COX regression analysis). **(G)** Kaplan–Meier curves of TCGA LIHC cohort. **(H)** C1QC+ TAMs independent of clinical features by using multivariate COX analyses. **(I)** Significantly top 1 inhibited and activated TF motifs during differentiation process colored by cell clusters.

Considering the immunosuppressive state of myeloid cells in tumor samples, the intrinsic functional subsets of the myeloid cells were further explored. Reclustering of the 5506 myeloid cells produced four clusters (**Figure 2A**). In contrast to the complex phenotypes of tumor-associated macrophages (TAMs) in the breast cancer and lung cancer TMEs (14, 22), the TAMs in HCC exhibited remarkable dichotomy (VCAN+ TAMs and C1QC+ TAMs) (**Figure 2A**). The coexistence of ‘‘classically activated’’ (M1) and ‘‘alternatively activated’’ (M2) macrophage signatures (23) in the VCAN+ TAMs and C1QC+ TAMs demonstrated the limitations of the M1 and M2 polarization model *in vitro* (**Supplementary Figures 2A, B**). Specifically, VCAN+ TAMs highly expressed myeloid-derived suppressor cell (MDSC)-associated genes, S100A family genes, VCAN, and FCN1 (**Figure 2C**) (24). In contrast, C1QC+ TAMs expressed the classical TAM-associated genes C1QA, C1QB, C1QC, APOE, and TREM2, which were previously reported in lung cancer (**Figure 2C**) (23). Notably, C1QC+ TAMs were specifically distributed in tumor tissue (**Figure 2A**). Moreover, the transcriptomes of the VCAN+ TAMs and C1QC+ TAMs showed gradual differences (**Figure 2C**), suggesting that VCAN+ TAMs were reprogrammed into C1QC+ TAMs in the tumor region. Following pseudotime trajectory analysis using Monocle2 to explore the potential transition between VCAN+ TAMs and C1QC+ TAMs, we observed that VCAN+ TAMs developed into C1QC+ TAMs with higher pseudotime scores, meaning that the C1QC+ TAMs were more mature and differentiated TAMs (**Figure 2B**), which confirmed that VCAN+ TAMs were reprogrammed into C1QC+ TAMs.

Pseudotime analysis was then conducted to investigate the dynamic changes in M1 and M2 scores in TAMs. The M2 score significantly increased with increasing pseudotime (**Figure 2D**). In this differentiation process, the coinhibitors HAVCR2, LAIR1, LGALS9, and CD276 were all upregulated, while the coactivators TNFSF14, CD48, and CD44 were downregulated (**Figure 2E**). These results suggested that VCAN+ TAMs undergo M2-like polarization in the tumor region and further exert immunosuppressive functions. Pathway activity in both TAM populations was analyzed by gene set variation analysis (GSVA), revealing strong enrichment of tumor vasculature, tumor angiogenesis, and extracellular matrix (ECM) regulator pathways in C1QC+ TAMs, while complement activation and cytokine pathways were significantly enriched in VCAN+ TAMs (**Supplementary Figure 3A**). Strikingly, the TGFB1 signaling, WNT signaling, and liver cancer metastasis pathways were specifically enriched in C1QC+ TAMs, suggesting that the cells play a protumorigenic and prometastatic role in HCC (**Supplementary Figure 3A**). The utility of the cell types identified in this study was evaluated with CIBERSORTx in samples from the TCGA LIHC cohort (16). Strikingly, only the accumulation of C1QC+ TAMs was related to poorer overall survival (OS) (**Figures 2F, G**). We also observed that the C1QC+ TAMs were independent of other clinical features, including age, sex, grade, and TNM stage, via multivariate Cox analyses, suggesting their independent prognostic value (**Figure 2H**). Through SCENIC analysis, we found that the activity of FOSL2 was downregulated, while activation of MAF motifs was responsible for the M2 polarization, in line with the gene expression patterns (**Figure 2I**; **Supplementary Figure 3B**). These results provide potential therapeutic targets for reversing the immunosuppressive microenvironment.

### Regulatory DCs exhibit an immune-suppressive phenotype in HCC

**Figure 3:**
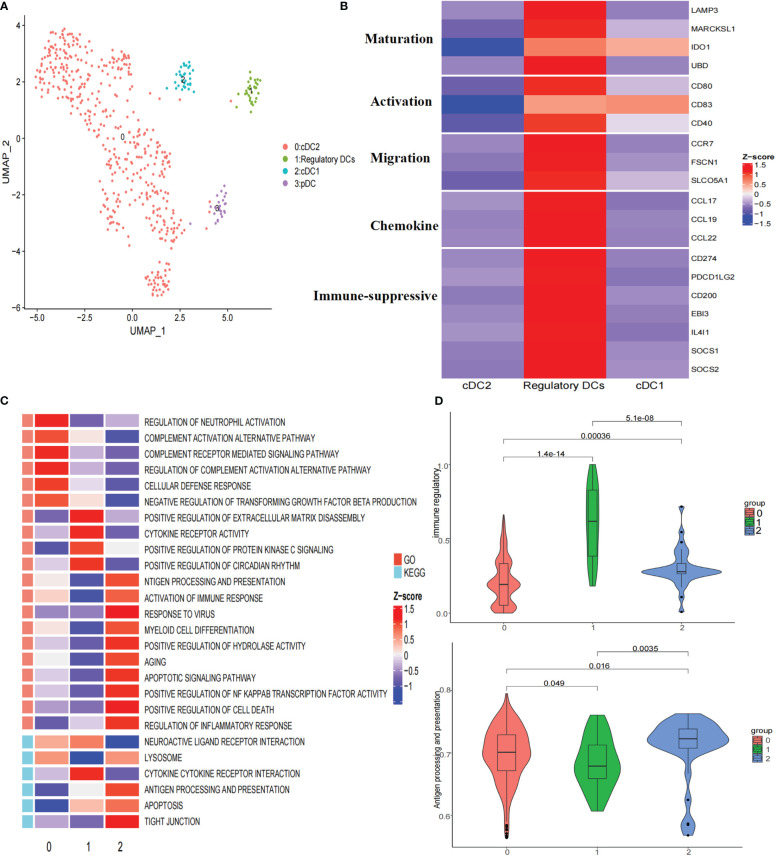
Re-clustering of DC cells. **(A)** UMAP plot of dendritic cells grouped into 4 cell types. **(B)** Heatmap showed the expression of maturation, activation, migration, chemokine, and immune suppressive molecules associated in three DCs clusters. **(C)** Heatmap showed the selected significant enrichment of GO and KEGG terms of three DCs clusters. **(D)** Violin plots showed the immune regulatory and antigen processing and presentation scores of three DCs clusters.

**Figure 4:**
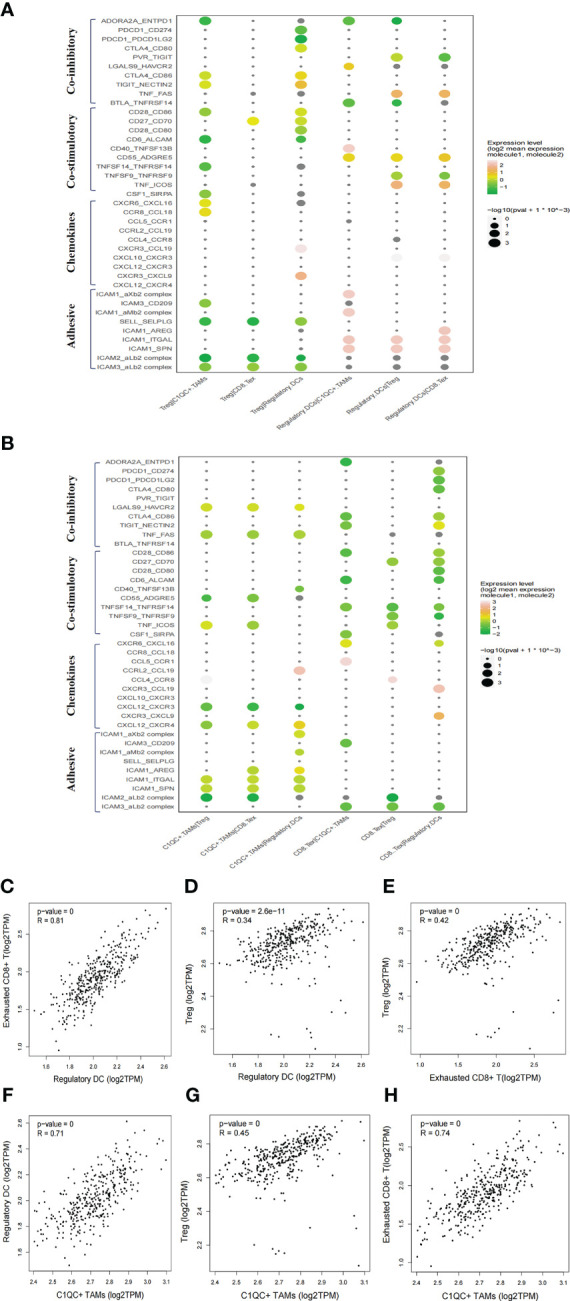
Intercellular interactions among C1QC+ TAMs, regulatory DC, Treg, and exhausted CD8+ T cells via co-inhibitory, co-stimulatory, chemokines or adhesive connection. **(A)** Intercellular interactions among Treg, regulatory DC, and other immune suppressive cells. **(B)** Intercellular interactions among C1QC+ TAMs, exhausted CD8+ T, and other immune suppressive cells. **(C–H)** Correlation between C1QC+ TAMs, regulatory DC, Treg, and exhausted CD8+ T cells subgroups in TCGA LIHC cohort.

To further explore the heterogeneity of dendritic cells (DCs), these cells were reclustered, resulting in four clusters: one cluster of plasmacytoid dendritic cells (pDCs), two clusters of conventional dendritic cells (cDCs), and one cluster of regulatory DCs (**Figure 3A**; **Supplementary Figure 4A**). Subcluster 3 highly expressed LILRA4, GZMB, and IL3RA, representing pDCs. Subcluster 2 highly expressed CLEC9A, CADM1, and XCR1, corresponding to cDC1s. Subcluster 0 highly expressed CD1C, FCER1A, and CLEC10A, representing cDC2s (**Supplementary Figure 4A**). Notably, subcluster 1 highly expressed the maturation markers LAMP3, MARCKSL1, IDO1, and UBD; activation markers CD80, CD83, and CD40; migration markers CCR7, FSCN1, and SLCO5A1; and immune-suppressive markers CD274, PDCD1LG2, CD200, EBI3, IDO1, IL4I1, SOCS1, and SOCS2 (**Figure 3B**), indicating that this subcluster closely resembled the ‘‘mregDCs’’ described by Maier (25). Regulatory DCs were significantly distributed in tumor tissues (**Supplementary Figure 4B**). It has been shown that cDCs can be educated towards a regulatory DC phenotype with immune suppressive functions by the TME (26). Analysis of signaling pathways by GSVA revealed that the term ‘activation of immune response’ was downregulated in regulatory DCs, which was consistent with this cluster showing upregulated expression of a subset of immune-suppressive genes and the highest immune regulatory score (**Figures 3B–D**). In addition, cytokine−cytokine receptor interactions were upregulated in regulatory DCs (**Figure 3C**). We found that these cells expressed various cytokines, including CCL17, CCL19, and CCL22 (**Figure 3B**). Previous studies have demonstrated that CCL19 has a strong ability to recruit regulatory T (Treg) cells through binding to CXCR3 (27). Moreover, the regulatory DC signature was positively correlated with the Treg cell signature in the TCGA LIHC cohort (**Figure 4D**). All these results suggested that regulatory DCs could directly inhibit CD8+ T cells through immunosuppressive molecules or indirectly inhibit CD8+ T cells by recruiting Treg cells into the tumor region.

To characterize the developmental origins of regulatory DCs, we applied the Monocle2 algorithm and observed that regulatory DCs might develop from cDC1s or cDC2s (**Supplementary Figure 4C**). Combined with their antigen presentation and immune regulatory scores (**Figure 3D**), these data suggested that cDCs might be converted into regulatory DCs with decreased antigen-presenting capacity and increased immune-suppressive ability in the tumor region.

### Immunosuppressive niche of the HCC TME

We then explored the potential functional subtypes of the T cells, which were grouped into 12 clusters based on marker gene expression (**Supplementary Figure 5A**). Given antitumor capabilities of CD8+ T cells, we performed pseudotime trajectory analysis to reveal the underlying evolution of these cells. Interestingly, we observed a gradual transition of CD8+ T cells towards an exhausted status (**Supplementary Figure 5B**). Exhausted CD8+ T cells in cell cluster 4 showed upregulated LAG3, PDCD1 (PD-1) and TIGIT (**Supplementary Figure 5C**). To explore the cellular communication network in HCC, we examined potential ligand−receptor binding pairs among different cell clusters derived from HCC tumors using CellPhoneDB software. We observed intensive cellular coinhibitory, costimulatory, chemokine and adhesion interactions among C1QC+ TAMs, regulatory DCs, Treg cell, and exhausted CD8+ T cells (CD8+ Tex_C3) that fostered an immunosuppressive niche (**Figures 4A, B**). Regulatory DCs had high expression of CD80/CD86, ADORA2A, and CD70, which showed ligand–receptor binding to CTLA4/CD28, ENTPD1, and CD27 on Treg cells, respectively, suggesting a potential interaction between regulatory DCs and Treg cells (**Figure 4A**). Regulatory DCs were also predicted to interact with Treg cells through CCL19-CXCR3 and CXCL10-CXCR3, which are known for recruiting Treg cells into tumor tissue (**Figure 4A**) (27). Regulatory DCs were also predicted to interact with exhausted CD8+ T cells through the classical immune-suppressive pathway and the TIGIT-PVR/NECTIN2 (NECTIN2, also called PVRL2) axis, a nonclassical immune-suppressive pathway involved in the suppression of antitumor responses (**Figure 4B**). Potential ligand–receptor interactions were observed between C1QC+ TAMs and Treg cells, including those of chemokines (CCL18-CCR8) and adhesion molecules (ICAM1-ITGAL and SELPLG-SELL), which promote the immune-suppressive activity of Treg cells in the TME (**Figures 4A, B**) (28). C1QC+ TAMs were also predicted to interact with exhausted CD8+ T cells through adhesion molecules (ICAM1-ITGAL and ICAM1-AREG) and immune molecules (HAVCR2-LGALS9) (**Figure 4B**), which are well-known to promote CD8+ T cell exhaustion in the TME (29). Consistently, we observed significant correlations of gene signatures among regulatory DCs, Treg cells, exhausted CD8+ T cells, and C1QC+ TAMs in an independent TCGA LIHC cohort (n = 369) (**Figures 4C–H**). We explored the potential cellular communication network between immune-suppressive niche cells (regulatory DCs, Treg cells, exhausted CD8+ T cells, and C1QC+ TAMs) and malignant cells. TIGIT-PVR/PVRL2 interactions were observed between HCC cells and Treg cells or exhausted CD8+ T cells (**Supplementary Figure 6A**). These findings suggest that intensive cellular crosstalk among immune-suppressive cell types plays a vital role in maintaining TME homoeostasis in HCC.

### The TIGIT-PVR/PVRL2 axis is a potential immunotherapeutic target in HCC patients

**Figure 5:**
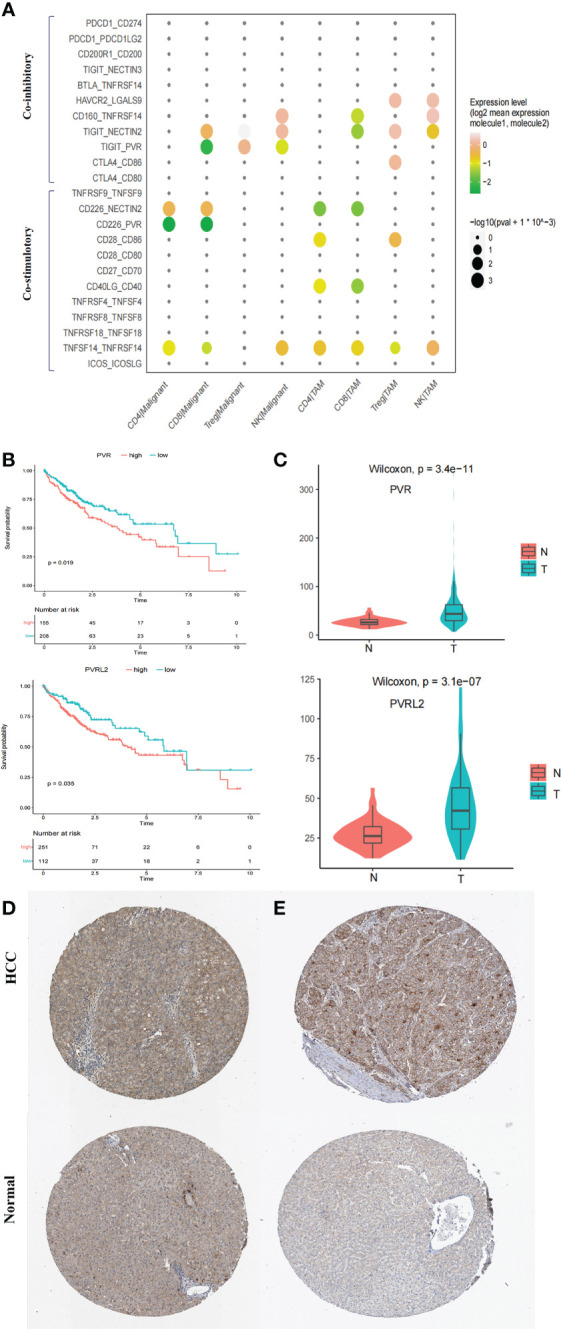
Immune checkpoint analysis in TME of HCC. **(A)** Intercellular interactions between tumor-infiltrating immune cells (CD4 T, CD8 T, Treg, and NK) and complementary antigen-presenting cells (APCs) (macrophages and tumor cells) via co-inhibitory and co-stimulatory. **(B)** High expressions of PVR and PVRL2 were significant associated with poorer OS, respectively. Log-rank test (two-sided). **(C)** PVR and PVRL2 was significantly upregulated in HCC tumor as compared to the non-tumorous livers in TCGA cohort. **(D)** IHC showed that PVR was significantly upregulated in HCC tumor tissue compared with the normal livers in Human Protein Atlas database. **(E)** IHC showed that PVRL2 was significantly upregulated in HCC tumor tissue compared with the normal livers in Human Protein Atlas database.

ICIs have reshaped cancer therapy. However, their efficacy and objective response rates are still unsatisfactory, indicating that our insufficient understanding of the immune microenvironment in HCC hinders effective drug development. We thus conducted systemic immune checkpoint analysis with both tumour-infiltrating immune cells (CD4 T, CD8 T, Treg, and natural killer (NK) cells) and complementary antigen-presenting cells (APCs) (macrophages, DCs, and tumour cells). The relative contribution of coinhibitory and costimulatory checkpoints in shaping the immunosuppressive landscape of HCC was estimated. We identified that the TIGIT–PVR/PVRL2 axis provides a prominent coinhibitory signal in tumour-infiltrating immune cells and APCs. The classical immune-suppressive CTLA4-CD80/CD86 and PD-1 (PDCD1)-PD-L1/L2 (CD274/PDCD1LG2) pathways showed minimal involvement (**Figure 5A**; **Supplementary Figure 6B**). In addition, further investigation using the TCGA LIHC cohort showed that high expression of PVR and PVRL2 was significantly associated with poorer OS of HCC patients (**Figure 5B**). Furthermore, in the TCGA cohorts, PVR and PVRL2 were significantly upregulated in HCC tumour compared to non tumour liver samples (**Figure 5C**). Moreover, using immunohistochemistry (IHC) data from the Human Protein Atlas database, we demonstrated significant upregulation of PVR and PVRL2 in HCC tumour compared to normal liver samples (**Figures 5D, E**). These findings suggest that overexpression of PVR/PVRL2 might be important in generating an immunosuppressive landscape during HCC development.

**Figure 6:**
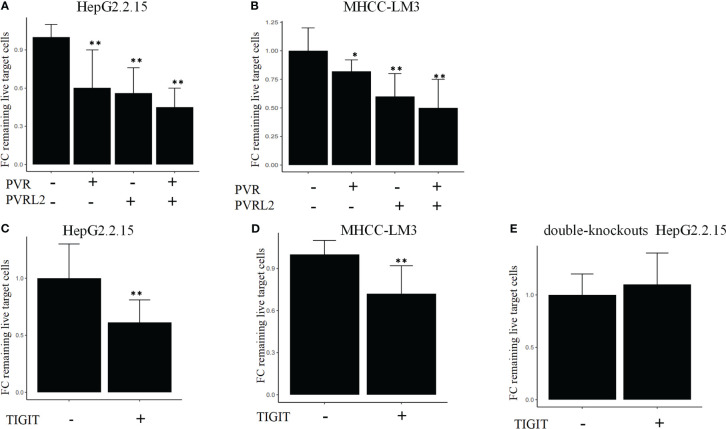
Blocking of the TIGIT-PVR/PVRL2 axis increases the lysis of HCC cell lines. **(A)** PBMC-mediated lysis, with subject to the blocking of PVR or PVRL2 on HCC cell lines HepG2.2.15. **(B)** PBMC-mediated lysis, with subject to the blocking of PVR or PVRL2 on HCC cell lines MHCC-LM3. **(C)** HepG2.2.15 was co-cultured with PBMCs with subject to the blocking of TIGIT. **(D)** MHCC-LM3 was co-cultured with PBMCs with subject to the blocking of TIGIT. **(E)** PVR and PVRL2 double-knockouts HepG2.2.15 was co-cultured with PBMCs with subject to the blocking of TIGIT. (N=5, *p ≤ 0.05; **p ≤ 0.001). Results are depicted as the mean ± SD fold changes (FC) of remaining live target cells, relative to the control without blocking antibodies.

**Figure 7:**
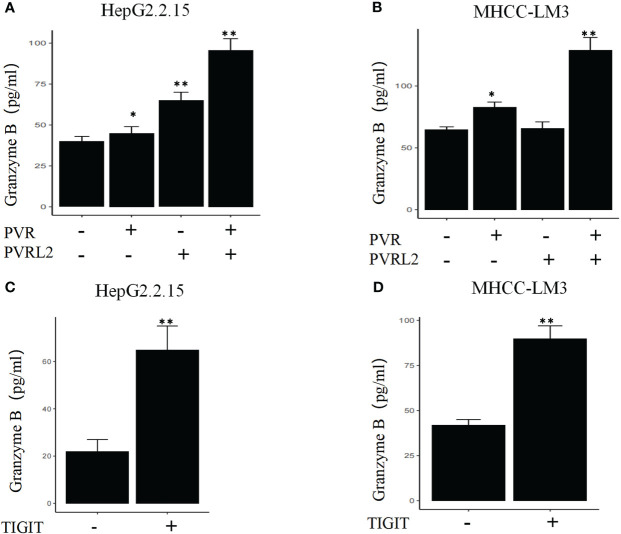
Blocking of the TIGIT-PVR/PVRL2 axis results in increased levels of Granzyme B secretion of immune cells. **(A)** HepG2.2.15 was co-cultured with PBMCs, and the secretion of GZMB was increased with subject to the blocking of PVR or PVRL2. **(B)** MHCC-LM3 was co-cultured with PBMCs, and the secretion of GZMB was increased with subject to the blocking of PVR or PVRL2. **(C)** HepG2.2.15 was co-cultured with PBMCs, and the secretion of GZMB was increased with subject to the blocking of TIGIT. **(D)** MHCC-LM3 was co-cultured with PBMCs, and the secretion of GZMB was increased with subject to the blocking of TIGIT. (N=5, *p ≤ 0.05; **p ≤ 0.001).

We continued to investigate the therapeutic potential of blocking novel immune checkpoint molecules PVR and PVRL2 for hepatocellular carcinoma through *in vitro* immune cell mediated cytotoxicity assays. In two HCC cell lines (HepG2.2.15 and MHCC-LM3), we found that blockade of PVR or PVRL2 increased immune cell-mediated lysis of tumour cell, with the combination group showing a higher enhancement effect (**Figures 6A, B**). At the same time, we also observed the changes of immune cell mediated killing effect of tumour cells by blocking TIGIT, and the results showed that the killing ability of the experimental group was significantly enhanced compared with the control group (**Figures 6C, D**). To further confirm that TIGIT blockade enhanced lysis in a manner dependent of target interactions with PVR/PVRL2, we generated PVR and PVRL2 double-knockouts for the cell line HepG2.2.15 using CRISPR/Cas9. We found that the enhanced cytotoxicity is eliminated (**Figure 6E**). The enhanced immune response was paralleled by the increased secretion of Granzyme B by immune cells in the cellular supernatant (**Figures 7A–D**). These results indicate that the TIGIT-PVR/PVRL2 axis is a potential target for immunotherapy of hepatocellular carcinoma.

## Discussion

HCC is a lethal malignancy, with more than 80% of patients being diagnosed at an advanced stage (3). Multiple TKIs (sorafenib, regorafenib, cabozantinib, and lenvatinib), ICIs (nivolumab and pembrolizumab), and vascular endothelial growth factor inhibitors (bevacizumab) have been used to systemically treat advanced HCC. Nevertheless, their efficacy is still unsatisfactory, and objective response rates are at most 25% (30). New therapy targets and/or combined therapy strategies are urgently needed, and such discoveries can be accelerated by a better understanding of the TME. Zhang et al. revealed the immune cells in the HCC TME have dynamic properties (12). Lu et al. further generated a landscape of the global cellular microenvironment of primary and metastatic HCCs (31). However, the analysis of functional state, clinical significance, and intercellular communication of immunosuppressive cells in the TME are still lacking. Additionally, the prominent coinhibitory signal in the immunosuppressive TME of HCC needs to be comprehensively evaluated. Herein, we generated a single-cell transcriptome atlas and revealed the functional state and clinical significance of immune-suppressive cell types. Moreover, we visualized the complex interaction network in the TME of HCC at single-cell resolution. Our present study provides strong evidence that the TIGIT-PVR/PVRL2 axis may represent a promising therapeutic target for HCC patients.

In contrast to the dynamic spectrum of TAM phenotypes in lung and breast cancer (14, 22), we identified that TAMs in HCC consisted of VCAN+ and C1QC+ TAMs, exhibiting remarkable dichotomy. Neither of the two clusters exactly matched the M1 or M2 phenotype, consistent with previous studies (14, 32). Rather, C1QC+ TAMs showed enrichment of tumor vasculature, tumor angiogenesis, and ECM regulator pathways, while complement activation and cytokine pathways were significantly enriched in VCAN+ TAMs. Notably, VCAN+ TAMs were reprogrammed into C1QC+ TAMs with upregulated coinhibitor and downregulated coactivator expression in the tumor region, suggesting key roles of C1QC+ TAMs in the tumorigenesis of HCC. Recently, depleting TAMs to promote antitumor immune responses has been revealed as a novel therapeutic approach. However, limited therapeutic benefit was observed in cancer patients treated with monotherapy, although TAMs were significantly depleted by CSF1R blockade (33). The heterogeneity and diverse functions of TAMs argue for more subset-specific strategies (34). In line with this, only the accumulation of C1QC+ TAMs was related to poorer OS. Our data suggest that specific depletion of C1QC+ TAMs could improve myeloid-targeted immunotherapy. As reported previously, tumor-associated myeloid cells are heterogeneous (14). We found that regulatory DCs exhibited immune regulatory and tolerogenic phenotypes with upregulated maturation, migration, and activation markers in line with those reported for regulatory DCs in multiple cancers recently (25, 35). Moreover, the regulatory DCs highly expressed the immune suppression-related genes CD274, PDCD1LG2, CD200, EBI3, IDO1, IL4I1, SOCS1, and SOCS2, showing a similar expression pattern to LAMP3+ DCs in lung cancer (25). We also observed that the regulatory DCs had a decreased antigen-presenting capacity and increased immune-suppressive ability in the tumor region. These results suggested that specifically depleting regulatory DCs or modulating these cells towards a normal phenotype may also be attractive therapeutic approaches for HCC.

We observed intensive cellular coinhibitory, costimulatory, chemokine and/or adhesion interactions among C1QC+ TAMs, regulatory DCs, Treg cells, and exhausted CD8+ T cells in HCC, suggesting that multiple immune suppressive cells foster an immunosuppressive niche in the TME of HCC. Indeed, regulatory DCs have a strong ability to recruit Treg cells through the interactions between CCL19-CXCR3 and CXCL10-CXCR3, which has also been demonstrated in colorectal cancers (27). In addition, regulatory DCs also highly express IDO1 in HCC, which can induce tumor-infiltrating Treg cell proliferation (36). On the other hand, Treg cells and regulatory DCs showed ligand–receptor binding through CTLA4-CD80/CD86, which regulate the maturation of tolerogenic DCs, consistent with studies in other cancers (37). The interactions between regulatory DCs and Treg cells could enhance the immune-suppressive effects of exhausted CD8+ T cells in HCC. Additionally, potential ligand–receptor interactions were observed between C1QC+ TAMs and Treg cells, including those of chemokines (CCL18-CCR8), which are well known to promote the immune-suppressive activity of Treg cells in the TME (28). C1QC+ TAMs interact with exhausted CD8+ T cells through immune regulation (HAVCR2-LGALS9) to promote CD8+ T cell exhaustion (29). These findings suggest that intensive cellular crosstalk among immune-suppressive cell types plays a vital role in maintaining TME homoeostasis in HCC. Thus, we propose that disrupting these intensive interactions could disrupt the balance of the TME and thus cure HCC.

The intratumor and intertumor heterogeneity of the malignant cells in HCC renders conventional chemotherapy or TKIs, which generally operate in a one-size-fits-all manner, ineffective in HCC. In this sense, immunotherapy, such as ICI therapy, instead of targeting certain specific tumor subclones is a more rational treatment option for advanced HCC patients. Furthermore, the relative contribution of coinhibitory and costimulatory checkpoints in shaping the immunosuppressive landscape of HCC was estimated. As the classical immune-suppressive CTLA4-CD80/CD86 and PD-1 (PDCD1)-PD-L1/L2 (CD274/PDCD1LG2) pathways were found to be minimally involved, they may have uncertain functions in treatment-naïve HCC patients. These findings could partially explain the unsatisfactory efficacy of anti-PD-1 antibodies and anti-CTLA4 antibodies in HCC treatment (38–40). Immune checkpoint therapeutic targets need to be further explored. Our findings suggested that the prominent coinhibitory signal of the TIGIT-PVR/PVRL2 axis is involved in HCC. Indeed, Zhang et al. mentioned potential interaction of LAMP3+ DCs and NK cells via TIGIT in the HCC TME (12). Ostroumov et al. reported that the expression of TIGIT is more reliable in identifying CD8+ T cell exhaustion than PD-1 (41). In addition, further investigation using the TCGA LIHC cohort in this study showed that high expression of PVR and PVRL2 was significantly associated with poorer OS of HCC patients. *In vitro*, antibody blockade of PVR or PVRL2 on HCC cell lines or TIGIT blockade on immune cells increased immune cell-mediated lysis of tumor cell. This enhanced immune response is paralleled by the increased secretion of Granzyme B by immune cells. These results demonstrated that the TIGIT-PVR/PVRL2 axis is a major coinhibitory immune checkpoint in human HCC. Hauke et al. demonstrated that PVR and PVRL2 act as prognostic markers and represent new therapeutic targets in acute myeloid leukemia *in vitro* (42). Additionally, ongoing clinical trials of anti-TIGIT antibodies in non-small-cell lung cancer (phase II CITYSCAPE trial) have shown encouraging treatment outcomes. It is highly anticipated that targeting the TIGIT-PVR/PVRL2 axis alone or in combination with other drugs may offer hope to HCC patients.

Our study had several limitations. First, cytotoxicity assays were performed by using HCC cell lines and healthy donor-derived immune cells. This approach differs from the TME *in vivo*. Blockade TIGIT-PVR/PVRL2 axis in an autologous setting using primary HCC cancer cells and the corresponding tumor-infiltrating lymphocytes should be further studied. Second, due to the limitations of the deconvolution algorithm, identifying the accurate proportion of VCAN+ TAMs and C1QC+ TAMs from bulk sequencing data is difficult. Last, batch effects, the main problems affecting bioinformatics analysis of scRNA data, could be present because fresh samples were required for the present study; however, we did attempt to remove batch effects with Seurat V3 in this study.

In summary, our study revealed the functional state, clinical significance, and intercellular communication of immunosuppressive cells in HCC at single-cell resolution. Furthermore, we found that immunosuppressive cells exhibit intensive potential intercellular crosstalk to foster an immunosuppressive niche in the HCC TME. Intriguingly, PVR/PVRL2, which are upregulated and unfavorable prognostic factors in HCC, interact with TIGIT and act as a prominent coinhibitory signal in the TME and was determined *in vitro*. Collectively, our scRNA-seq data suggested that targeting the TIGIT-PVR/PVRL2 axis with blocking antibodies might be a promising efficacious therapy strategy in HCC.

## Data availability statement

The data presented in the study are deposited in the Sequence Read Archive repository, accession number PRJNA947270.

## Ethics statement

The studies involving human participants were reviewed and approved by Human Ethics Committee of the First Hospital of Jilin University. The patients/participants provided their written informed consent to participate in this study.

## Author contributions

AL and BJ drafted the initial manuscript. BJ and YY collected clinical information. JL, RG, QZ, and BK assisted with the total experiments. BY, QMZ, XH, YL, and PZ were responsible for data analysis. YJ supervised the project and designed the experiments. All authors contributed to the article and approved the submitted version.

## Supplementary material

Supplementary Figure 1 Quality control of single cell RNA sequencing data and identifying major cell types. **(A)** Removal of the batch effect between batches. **(B)** Violoin plot showed the number of genes (nFeature), number of UMI (nCount) and percent of mitochondrial derived transcripts (percent.mt) per single cell in each sample. **(C)** UMAP plot showed the numbers of nFeature, nCount, and percent.mt per single cell. **(D)** Information of cells in each sample after quality control. **(E)** The fraction of cell types in each sample. **(F)** Heatmap of marker genes of every cell type. Shown are row z-score.

Supplementary Figure 2 M1 and M2 signatures in VCAN+ TAMs and C1QC+ TAMs. **(A)** M1 signatures in VCAN+ TAMs and C1QC+ TAMs. **(B)** M2 signatures in VCAN+ TAMs and C1QC+ TAMs.

Supplementary Figure 3
**(A)** Differential pathway enriched in VCAN+ TAMs and C1QC+ TAMs by GSVA. **(B)** Expressions of top 1 activated or inhibited TF motifs in the differentiation process colored by cell clusters.

Supplementary Figure 4
**(A)** Heatmap of DEGs between three different DC clusters. **(B)** UMAP plot of DC cells colored by cell origins. **(C)** Pseudotime trajectory analysis of three DC clusters.

Supplementary Figure 5
**(A)** UMAP plot of T cells grouped into 12 cell types. **(B)** Pseudotime trajectory analysis of CD8+ T cell clusters. **C)** Representative markers indicate immune suppressive and exhausted status.

Supplementary Figure 6
**(A)** Potential cellular communication network between Immune-suppressive niche cells (regulatory DC, Treg, exhausted CD8+ T cells, and C1QC+ TAMs) and malignant. **(B)** Intercellular interactions between tumor-infiltrating immune cells (CD4 T, CD8 T, Treg, and NK) and DC cells.
