# Supplementary figures and images for: Single-cell RNA sequencing highlights the role of PVR/PVRL2 in the immunosuppressive tumour microenvironment in hepatocellular carcinoma
Source: Front Immunol. 2023 Jun 13;14:1164448. doi: 10.3389/fimmu.2023.1164448 (PMC10293927; doi:10.3389/fimmu.2023.1164448)

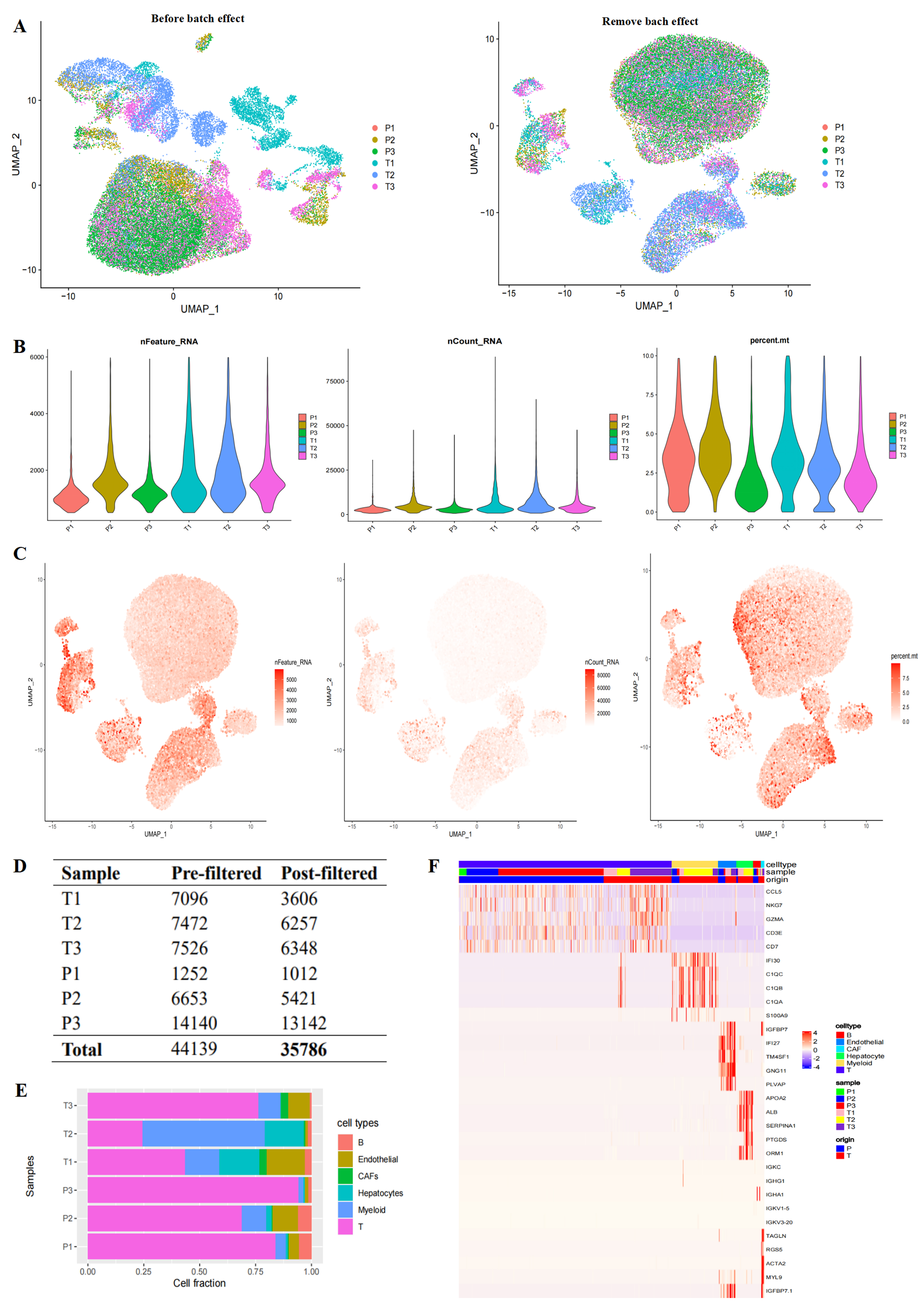

Supplement: Supplementary Figure 1 — Quality control of single cell RNA sequencing data and identifying major cell types. (A) Removal of the batch effect between batches. (B) Violoin plot showed the number of genes (nFeature), number of UMI (nCount) and percent of mitochondrial derived transcripts (percent.mt) per single cell in each sample. (C) UMAP plot showed the numbers of nFeature, nCount, and percent.mt per single cell. (D) Information of cells in each sample after quality control. (E) The fraction of cell types in each sample. (F) Heatmap of marker genes of every cell type. Shown are row z-score. [file Image_1.jpeg]

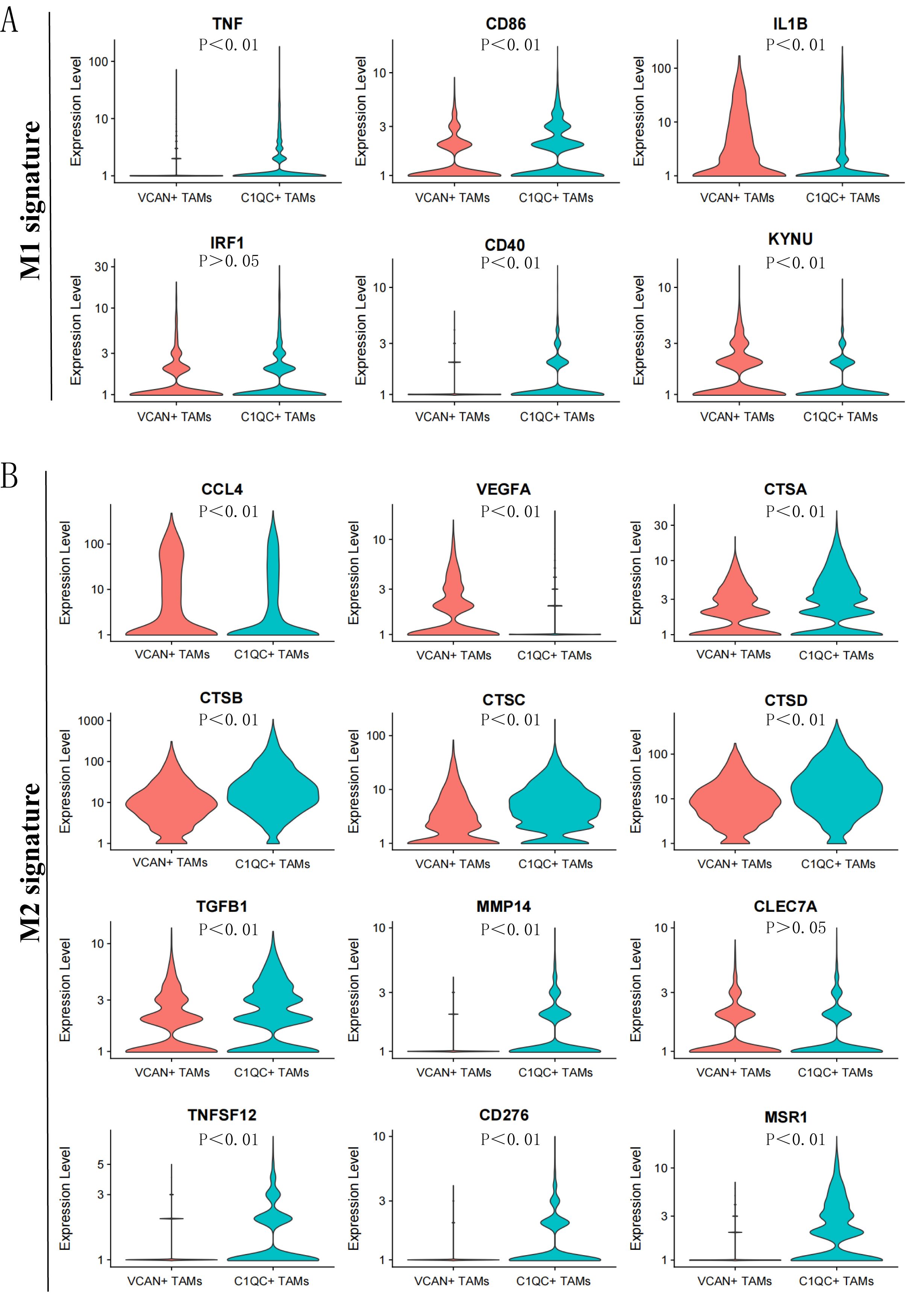

Supplement: Supplementary Figure 2 — M1 and M2 signatures in VCAN+ TAMs and C1QC+ TAMs. (A) M1 signatures in VCAN+ TAMs and C1QC+ TAMs. (B) M2 signatures in VCAN+ TAMs and C1QC+ TAMs. [file Image_2.jpeg]

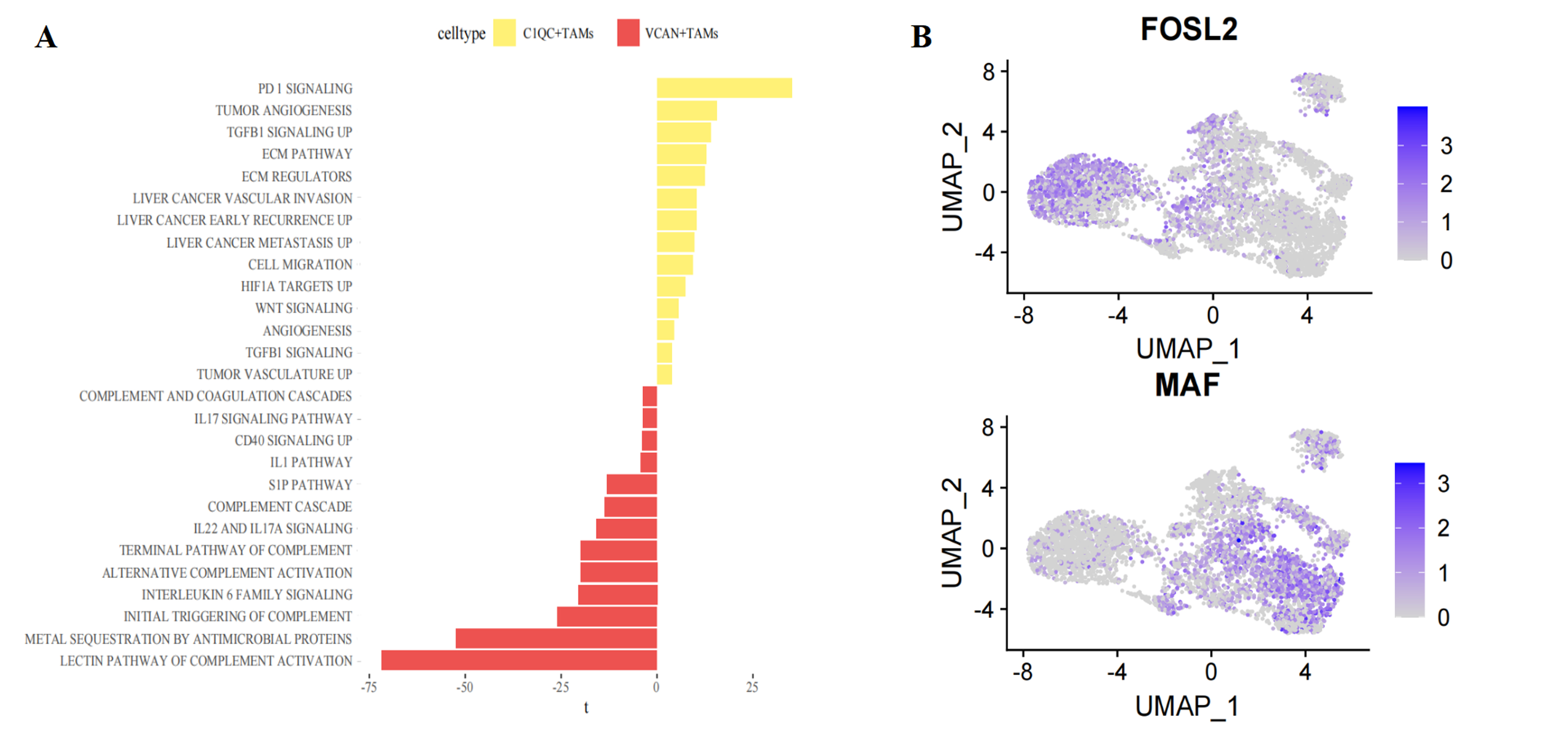

Supplement: Supplementary Figure 3 — (A) Differential pathway enriched in VCAN+ TAMs and C1QC+ TAMs by GSVA. (B) Expressions of top 1 activated or inhibited TF motifs in the differentiation process colored by cell clusters. [file Image_3.jpeg]

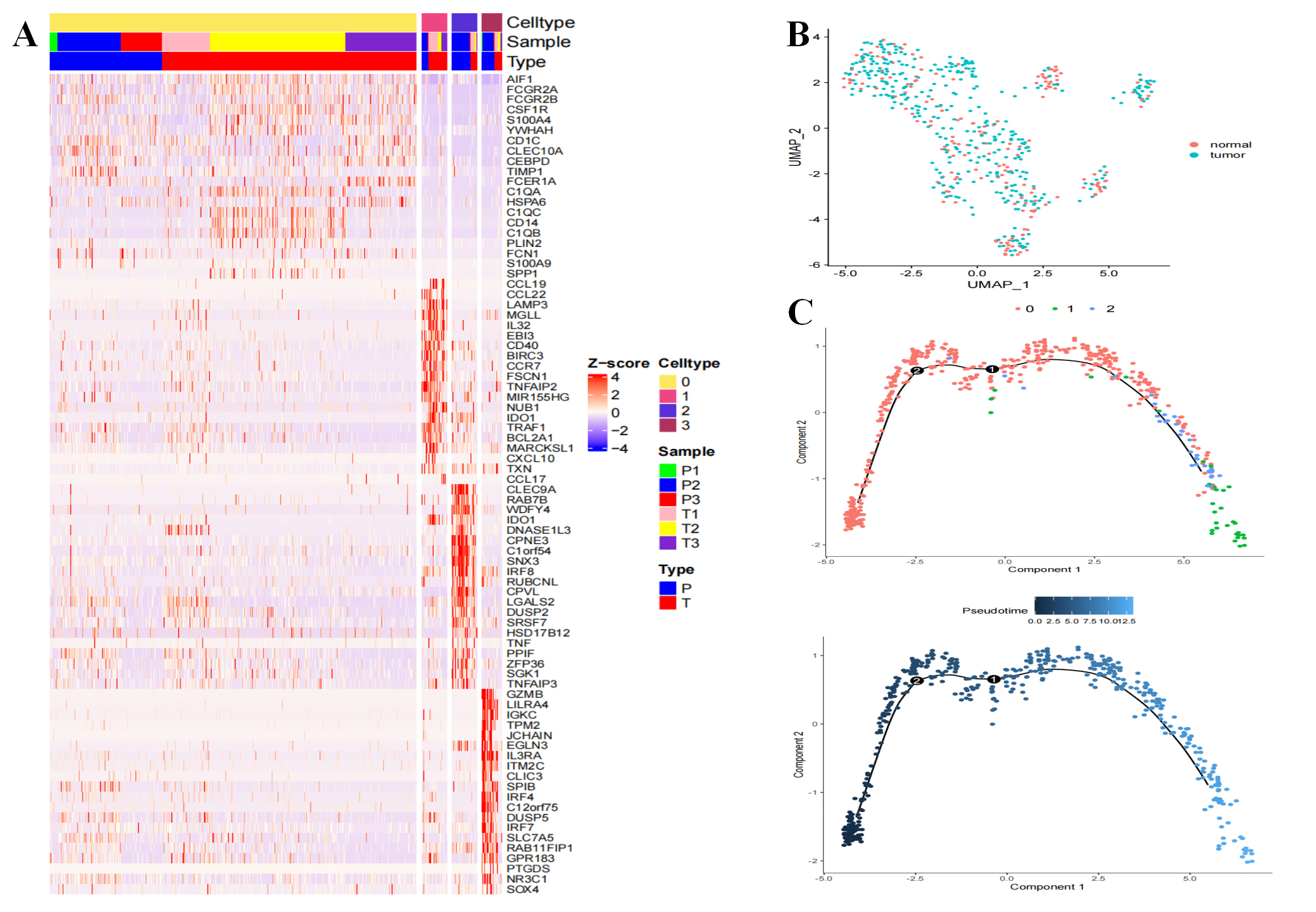

Supplement: Supplementary Figure 4 — (A) Heatmap of DEGs between three different DC clusters. (B) UMAP plot of DC cells colored by cell origins. (C) Pseudotime trajectory analysis of three DC clusters. [file Image_4.jpeg]

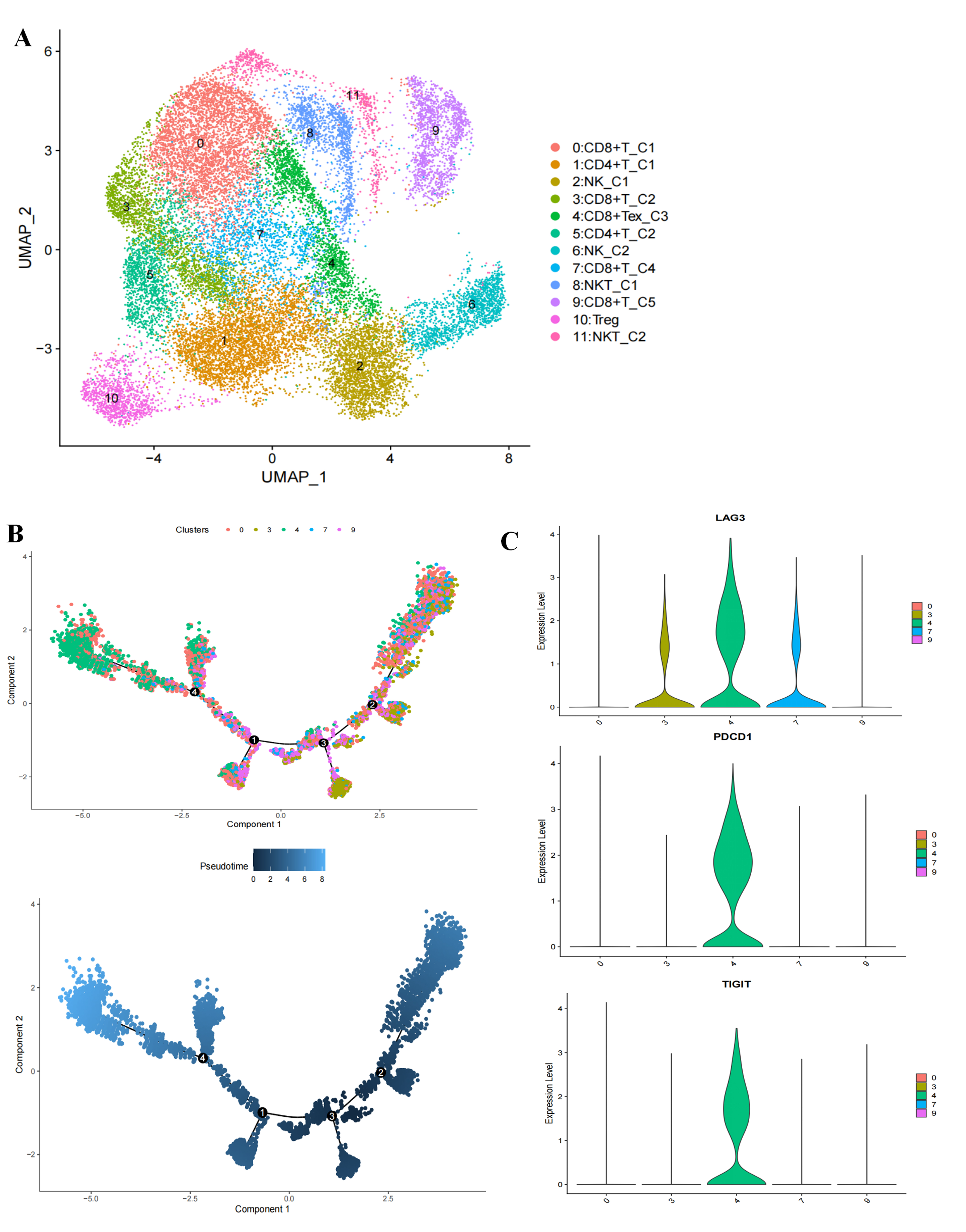

Supplement: Supplementary Figure 5 — (A) UMAP plot of T cells grouped into 12 cell types. (B) Pseudotime trajectory analysis of CD8+ T cell clusters. C) Representative markers indicate immune suppressive and exhausted status. [file Image_5.jpeg]

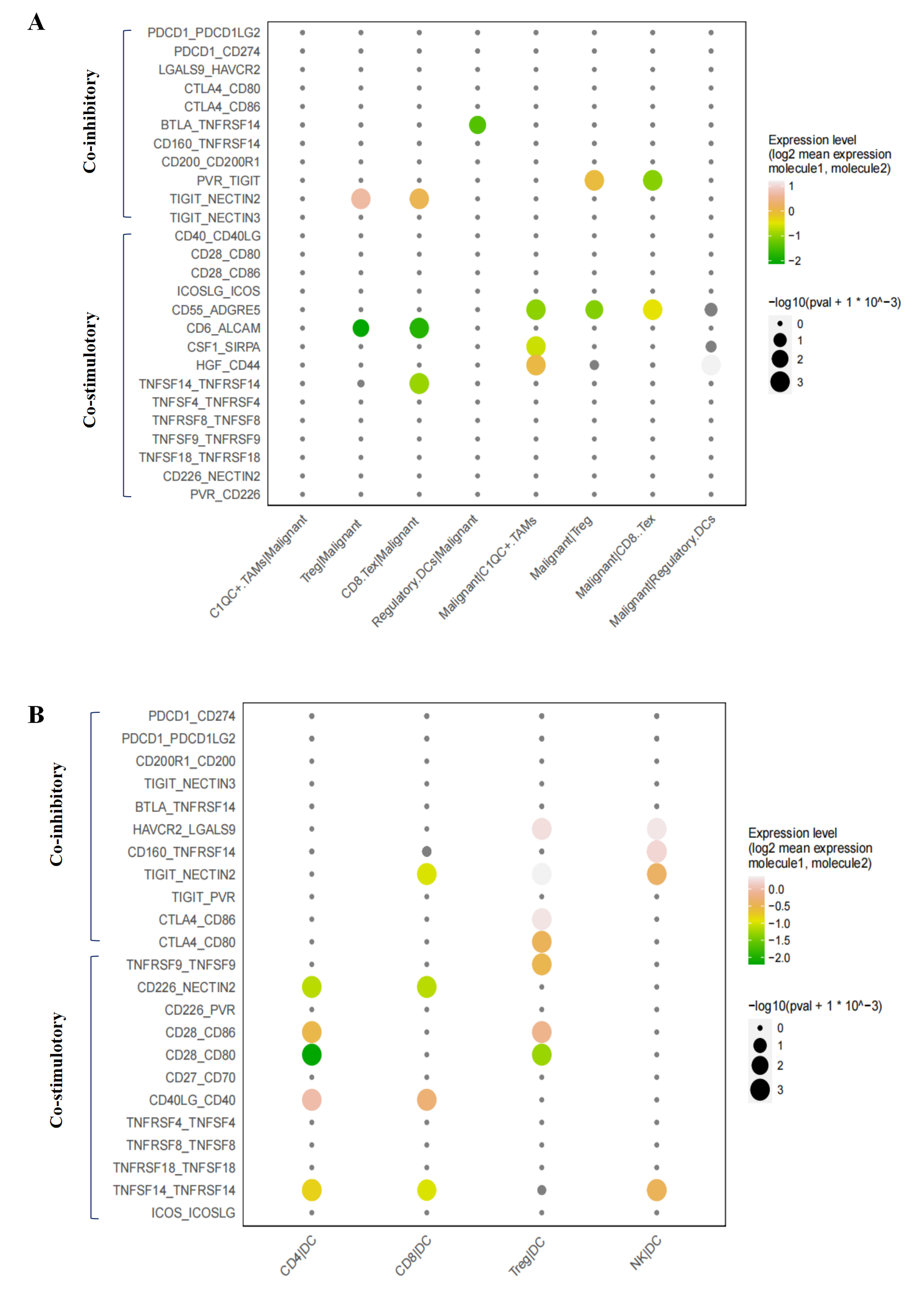

Supplement: Supplementary Figure 6 — (A) Potential cellular communication network between Immune-suppressive niche cells (regulatory DC, Treg, exhausted CD8+ T cells, and C1QC+ TAMs) and malignant. (B) Intercellular interactions between tumor-infiltrating immune cells (CD4 T, CD8 T, Treg, and NK) and DC cells. [file Image_6.jpeg]
