# Supplementary material for: Single-cell RNA sequencing highlights the role of PVR/PVRL2 in the immunosuppressive tumour microenvironment in hepatocellular carcinoma
Source: Front Immunol. 2023 Jun 13;14:1164448. doi: 10.3389/fimmu.2023.1164448 (PMC10293927; doi:10.3389/fimmu.2023.1164448)
Supplement: Supplementary file 7 [file Table_1.doc]

Supplementary Table 1. Clinical characteristics of 3 HCC patients in this study

| Case number | P1 | P2 | P3 |
| --- | --- | --- | --- |
| Age (years) | 54 | 55 | 61 |
| Gender | M | M | F |
| No. of tumor nodules | 1 | 1 | 1 |
| Tumor size (cm) | 3.6*3.5*3 | 12*10*7 | 5.8*4.7*3 |
| Cellular differentiation | Moderate | Low | Low |
| Venous invasion | Present | Present | Present |
| Liver invasion | Absent | Present | Present |
| Background liver | NS | Cirrhosis | Cirrhosis |
| HBV status | Positive | Positive | Positive |
| HCV status | Negative | Negative | Negative |
| HBV DNA (IU/ml) | 8.13e4 | 2.14e4 | 1.15e8 |
| Hyperlipidemia | No | No | No |
| Alcoholism | No | No | No |

P, patient; M, male; F, female; NS, non-specific changes
